# Artificial intelligence-driven precision management of *Helicobacter pylori*: an integrated framework from endoscopy and multi-omics to vaccine design and digital care

**DOI:** 10.3389/fcimb.2026.1999727

**Published:** 2026-10-29

**Authors:** Minglin Zhang, Nuonan Lv, Yanghui Cao, Lingqiao Lou, Ting Cai, Jiaxing Zhu, Yingting Xu, Xuelin Zhao, Zhiyuan Ma, Xuemei Liu

**Affiliations:** 1 Department of Gastroenterology, Digestive Disease Hospital, Affiliated Hospital of Zunyi Medical University, Zunyi, Guizhou, China; 2 Department of Pharmacy, The Third Xiangya Hospital, Central South University, Changsha, Hunan, China; 3 Department of Gastroenterology, Hunan Provincial People’s Hospital, The First Affiliated Hospital of Hunan Normal University, Changsha, Hunan, China; 4 Hunan Provincial University Key Laboratory of the Fundamental and Clinical Research on Neurodegenerative Diseases, Changsha Medical University, Changsha, Hunan, China; 5 Department of Gastroenterology, The Third Xiangya Hospital, Central South University, Changsha, Hunan, China

**Keywords:** artificial intelligence, drug resistance prediction, endoscopic diagnosis, Helicobacter pylori, multimodal fusion, vaccine design

## Abstract

*Helicobacter pylori* (*H. pylori*) infection, affecting half of the world’s population, is the principal cause of peptic ulcer disease and gastric cancer, yet its clinical management is increasingly undermined by the rapidly escalating global burden of antibiotic resistance, the suboptimal accuracy of conventional diagnostic methods, and the declining efficacy of empirical therapeutic regimens. Artificial intelligence (AI) is rapidly reshaping this landscape, but existing research remains fragmented across single modalities. We propose a conceptual five-module framework that integrates AI approaches across the *H. pylori* care continuum: (1) global disease burden and the evolutionary dynamics of *H. pylori* drug resistance; (2) the application of multimodal AI, from convolutional neural networks to vision-language foundation models, for endoscopic early screening, pathological identification, and functional assessment; (3) the harnessing of machine learning (ML) on genomic and electronic health record data to decipher resistance mechanisms and generate personalized eradication regimens; (4) AI-driven vaccine target discovery and generative design, spanning reverse vaccinology to *de novo* immunogen creation; and (5) a digital closed-loop patient management platform that leverages new media and reinforcement learning to enhance adherence and eradication rates. We further delineate the core challenges of data bias, cross-scenario generalization, and the critical absence of prospective randomized evidence, and propose a future direction anchored in multimodal foundation models, federated learning, and continuous AI quality improvement. Rather than an established clinical pathway, this framework is proposed as a conceptual blueprint that, once prospectively validated, could upgrade AI-enabled *H. pylori* care from isolated image recognition toward an end-to-end “perception–reasoning–intervention” system, offering a blueprint for precision prevention and control.

## 1 Introduction

*Helicobacter pylori* (*H. pylori*) is a spiral-shaped, Gram-negative bacterium that colonizes the gastric mucosa and causes mucosal injury. It is intimately linked to a spectrum of gastrointestinal diseases, including chronic gastritis, peptic ulcer disease, gastric cancer, and gastric mucosa-associated lymphoid tissue (MALT) lymphoma (Malfertheiner et al., 2014). Infecting approximately 50% of the global population, *H. pylori* has been designated a Group 1 carcinogen by the International Agency for Research on Cancer (IARC) of the World Health Organization and is associated with roughly 90% of gastric cancer cases worldwide (Malfertheiner et al., 2022). Eradication of *H. pylori* can significantly arrest the progression of gastric precancerous lesions—most notably intestinal metaplasia and chronic atrophic gastritis (CAG)—and even promote their regression (Zhu et al., 2023). Nevertheless, the diagnosis and treatment of *H. pylori* infection continue to face formidable challenges. The global disease burden is reflected not only in its high prevalence but also in its severe clinical sequelae. Several clinical bottlenecks persist in *H. pylori* management: endoscopic detection of precancerous lesions and early gastric cancer attributable to *H. pylori* infection remains challenging for endoscopists, with an endoscopic diagnostic accuracy for *H. pylori* infection of only about 70% (Luo et al., 2022). Antibiotic resistance rates are escalating steadily; clarithromycin resistance, in particular, has emerged as a predominant driver of treatment failure worldwide, eroding the success rates of traditional eradication regimens and compounding the burden and failure risk of rescue therapies following unsuccessful empirical treatment (Lu et al., 2026; Matei et al., 2026; Villegas et al., 2025).

In recent years, artificial intelligence (AI) has demonstrated immense potential in medicine, catalyzing a paradigm shift from “computer-aided diagnosis (CAD)” to “AI-powered precision diagnosis and treatment strategies.” In gastroenterology, AI has been widely deployed for endoscopic analysis, cancer detection, liver fibrosis assessment, and disease differentiation (Le Berre et al., 2020). A deep learning-based AI diagnostic system for *H. pylori* infection (HOPE AI), leveraging large-scale endoscopic images and videos, has achieved outstanding performance across multiple validation sets, delivering significantly higher diagnostic sensitivity than senior endoscopists (Hu et al., 2025). AI models have also shown high accuracy in identifying *H. pylori* in Warthin-Starry (W-S) silver-stained histopathological images (Liscia et al., 2022). Furthermore, AI exhibits considerable promise in the diagnosis and risk stratification of CAG (Tao et al., 2024). By integrating imaging, pathological, molecular, and clinical phenotype data, multimodal AI technologies have markedly advanced diagnostic and therapeutic decision-making for gastrointestinal tumors, functional gastrointestinal disorders, and liver diseases, enhancing both early diagnostic accuracy and the efficiency of personalized treatment (Wu et al., 2025). The empowerment of *H. pylori* care by AI now extends far beyond image recognition and should be upgraded to a next-generation diagnostic and therapeutic strategy encompassing the entire “perception–reasoning–intervention” chain. AI not only assists clinicians in identifying *H. pylori* infection from endoscopic images but also provides personalized treatment recommendations and predicts therapeutic outcomes based on patient data (Higgins et al., 2025).

This review proposes a conceptual five-module framework that spans from molecular characterization to population-level care (**Figure 1**), while explicitly distinguishing applications already validated in clinical settings from those that remain experimental or represent future directions: (1) mapping the global disease burden and the evolutionary landscape of *H. pylori* drug resistance to reveal epidemiological trends and region-specific resistance challenges; (2) focusing on the fusion application of multimodal AI in endoscopic early screening, pathological identification, and functional assessment to enhance early diagnostic precision; (3) on this basis, harnessing AI to decipher molecular mechanisms of resistance and generate personalized eradication regimens, thereby enabling precision therapy; (4) simultaneously exploring AI-driven vaccine target discovery and vaccine design to counteract the spread of antimicrobial resistance; and (5) ultimately proposing a digital closed-loop management platform that supports multicenter collaboration under robust privacy protection, covering intelligent management of the entire continuum from diagnosis and treatment to long-term follow-up. Rather than a validated clinical pathway, this framework is proposed as a conceptual blueprint that could overcome the limitations of previous single-modality investigations and provide a new paradigm for the comprehensive, intelligent prevention and control of *H. pylori* infection.

**Figure 1:**
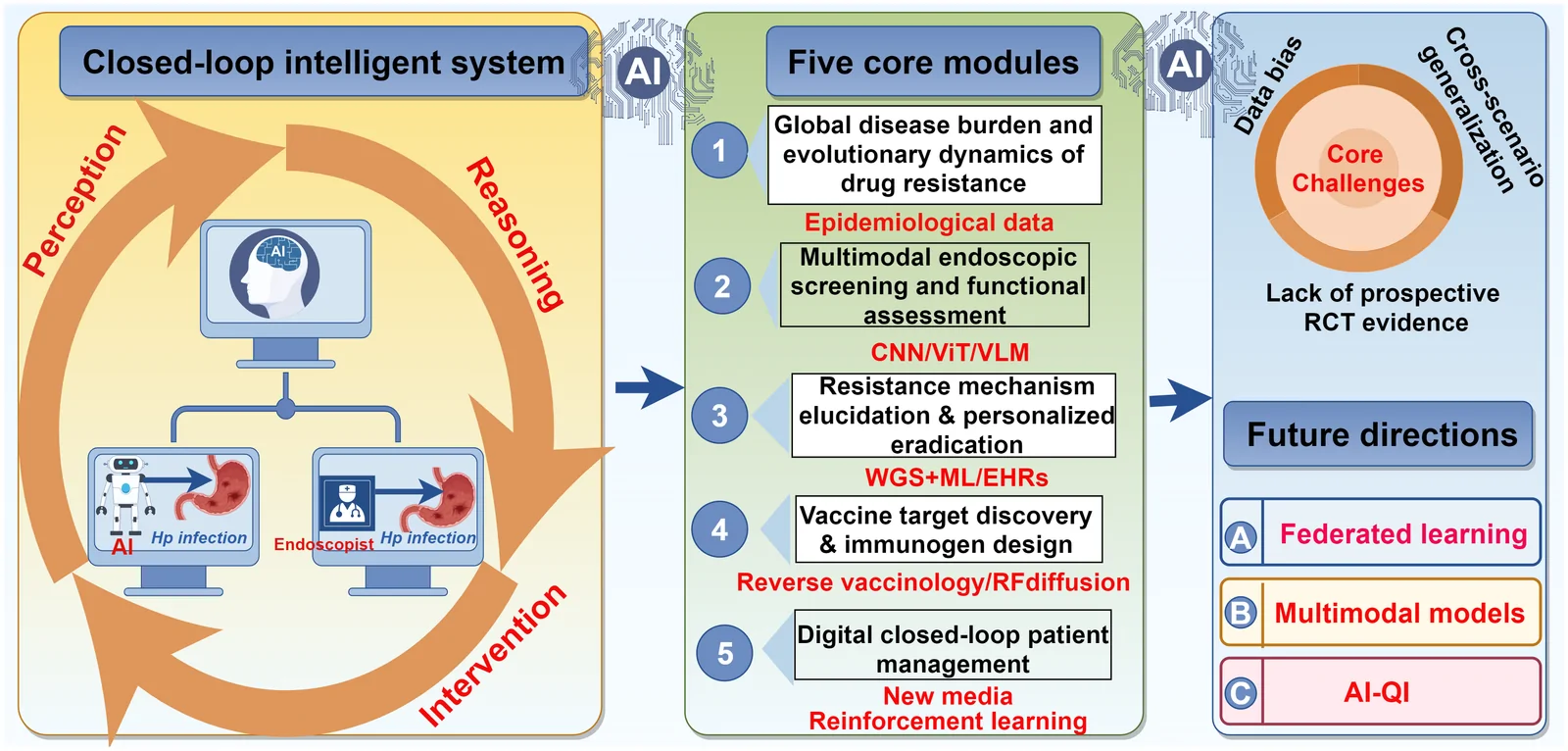
A proposed AI-driven precision-management framework for *H. pylori* infection. The figure depicts a conceptual, not yet clinically validated closed-loop intelligent system that integrates five core modules across perception, reasoning, and intervention: i) global disease burden and dynamic evolution of antimicrobial resistance; ii) multimodal endoscopic screening and functional assessment based on convolutional neural networks, vision transformers, and vision-language foundation models; iii) deciphering resistance mechanisms and developing individualized eradication regimens using whole-genome sequencing, machine learning, and electronic health records; iv) vaccine target discovery and immunogen design through reverse vaccinology and generative AI; and v) digital closed-loop patient management integrating new media and reinforcement learning. The figure additionally highlights key challenges (data bias, insufficient cross-scenario generalization, and the lack of prospective randomized controlled trial evidence) and future directions (federated learning, multimodal models, and continuous quality improvement through AI).

A literature search was conducted in PubMed/MEDLINE, Embase, and Web of Science. The search strategy combined pathogen-related terms (“*Helicobacter pylori*” or “*H. pylori*” or “Hp”) with artificial intelligence-related terms (“artificial intelligence” or “machine learning” or “deep learning” or “convolutional neural network” or “vision-language model” or “foundation model” or “generative AI” or “reinforcement learning”), as well as domain-specific terms related to endoscopy, antimicrobial resistance/genomics, vaccines, and digital/adherence interventions. Articles retrieved were selected based on methodological quality, clinical relevance, and scientific impact; where available, preference was given to meta-analyses, multicenter and prospective studies, externally validated models, and randomized controlled trials. Studies not directly related to *H. pylori* or AI, and conference abstracts, were excluded.

## 2 AI-assisted endoscopic diagnosis of *H. pylori* infection: from CNNs to vision-language large models

Endoscopic diagnosis of *H. pylori* infection has long relied on operator experience, with limited interobserver agreement, and poses significant challenges particularly in identifying previous infection (post-eradication) and CAG (Luo et al., 2022). In recent years, the application of AI in this field has undergone a paradigm shift from traditional convolutional neural network (CNN) classification to vision-language models (VLMs), achieving not only continuously improved diagnostic performance but also substantial progress in multicenter prospective validation and primary care implementation. Throughout this section we explicitly indicate the level of evidence of each study, distinguishing retrospective internal validation, external validation, prospective clinical validation, and randomized trials (**Table 1**).

**Table 1:** Maturity and evidence level of AI applications across the *H. pylori* care continuum (authors’ assessment).

Domain	Representative AI application	Highest available evidence level	AI-specific?	Estimated timeline to routine clinical integration
Endoscopic diagnosis	CNN/VLM-based detection of H. pylori infection status	External + prospective multicenter validation; benchmark RCTs exist for other upper-GI tasks (not H. pylori-specific)	Yes	≈1–3 years
Multimodal risk stratification	Fusion of endoscopy, pathology, and EHR data for gastric-cancer risk	Retrospective/internal; early multicenter validation	Yes	≈2–5 years
Drug-resistance prediction	ML on WGS/SNPs to predict antibiotic resistance	Research-grade; external genomic cohorts; proof-of-concept	Yes	≈3–7 years (limited by turnaround/cost)
Personalized regimen recommendation	isDQN-based AI-Clinician	Large retrospective + external validation; no RCT	Yes	≈3–7 years
Vaccine design	Reverse vaccinology/generative immunogen design	In silico and animal proof-of-concept	Yes	≈5–10 years
Adherence support (conventional)	Short-message/telephone/WeChat reminders	RCTs and meta-analyses	No	Already available
Adherence support (AI-driven)	RL-optimized messaging; AI voice cloning	Early studies; limited validation	Yes	≈2–5 years

### 2.1 Deep learning-based endoscopic identification of *H. pylori*: technical evolution

#### 2.1.1 Pioneering work: the first application of CNN to *H. pylori* endoscopic diagnosis

The application of AI to *H. pylori* endoscopy dates back to 2017, when Shichijo et al. trained a CNN on more than 30, 000 gastric endoscopic images and achieved a diagnostic accuracy of 87.7% (AUC 0.93) on an independent test set of 397 patients, significantly outperforming general endoscopists and reaching parity with experienced (board-certified) endoscopists, while requiring only about 3 minutes per diagnosis versus an average of 230 minutes for endoscopists; categorizing training images by gastric anatomical subsite further improved performance (Shichijo et al., 2017). A subsequent GoogLeNet-based model trained on 179 lesser-curvature images with data augmentation reached 86.7% sensitivity and specificity (AUC 0.956) (Itoh et al., 2018). Although small and mostly single-center retrospective studies, these early works established the fundamental paradigm of extracting *H. pylori*-related mucosal texture features from endoscopic images (**Table 2**).

**Table 2:** Summary of key studies on artificial intelligence-based endoscopic diagnosis of *H. pylori*.

Study	Year	Model architecture	Data scale	Main performance metrics	Key features
Shichijo et al. (Shichijo et al., 2017)	2017	CNN	>30, 000 images	Accuracy 87.7%, AUC 0.93	First CNN application; diagnosis significantly faster than endoscopists
Itoh et al. (Itoh et al., 2018)	2018	GoogLeNet	179 images	Sensitivity 86.7%, Specificity 86.7%, AUC 0.956	Data augmentation, small-sample training
Zheng et al. (Zheng et al., 2019)	2019	ResNet-50	Single-center	Accuracy, sensitivity, specificity all >90%	Performance close to direct detection methods; potential to replace biopsy
Nakashima et al. (Nakashima et al., 2020)	2020	LCI-CAD (CNN)	–	Accuracy 79%–84%	Combines linked color imaging; distinguishes current/past/uninfected
Yang et al. (Yang et al., 2024)	2024	EfficientNetB7 (multi-task)	6, 270 images	*H. pylori* accuracy 83.33%, AUC 0.91	Multi-task learning; simultaneous assessment of multiple lesions
Hu et al. (Hu et al., 2025)	2025	HOPE AI (ViT+LSTM+MIL)	6, 207 patients, >350, 000 images	Internal AUC 0.932; external AUC 0.855–0.971; sensitivity 85.7%	Multicenter multiple instance learning; significantly outperforms senior endoscopists
Sun et al. (Sun et al., 2025)	2025	MC-CLIP (VLM)	Pretrained on 2.42M image–text pairs	Overall accuracy 89.6%/86.6%; current infection sensitivity 91.4%	Vision–language model; automatic image selection; capsule endoscopy

More broadly, Hirasawa et al. showed that a CNN could detect gastric cancer from endoscopic images with 92.2% sensitivity within a very short time, establishing the “large-scale endoscopic images + deep learning” route that influenced subsequent *H. pylori* AI research; CNNs were thereafter shown to rapidly classify the three *H. pylori* infection states (positive, negative, post-eradication) from endoscopic images (Hirasawa et al., 2018; Shichijo et al., 2019).

#### 2.1.2 CNN architecture optimization and imaging modality expansion

With the rapid evolution of deep learning architectures, model performance for *H. pylori* endoscopy has continuously improved. A ResNet-50 model based on gastric endoscopic images assessed *H. pylori* infection with sensitivity, specificity, and accuracy all exceeding 90%, comparable to clinically recommended direct detection methods and holding promise as a replacement for gastric biopsy (Zheng et al., 2019). A linked color imaging-based CAD system (LCI-CAD) classified infection status into uninfected, current infection, and post-eradication with an accuracy of approximately 79%–84%, comparable to experienced endoscopists and superior to white-light-imaging CAD (Nakashima et al., 2020).

Further architectural refinement has also been explored. By integrating a CNN with concurrent spatial and channel squeeze-and-excitation (scSE) modules and combining multiple classifiers, the scSE-CatBoost model diagnosed *H. pylori* infection with 90% accuracy and 100% sensitivity (Lin et al., 2023a). A hybrid CNN (pretrained ShuffleNet for feature extraction, canonical correlation analysis for fusion, ReliefF feature selection, and a generalized additive model for classification) detected *H. pylori*-related atrophic gastritis with 98.2% accuracy, outperforming GoogLeNet and ResNet-50 (Yacob et al., 2023).

#### 2.1.3 Multi-task learning: from single diagnosis to comprehensive assessment

Traditional *H. pylori* endoscopic AI models have primarily focused on a single task (e.g., infection-status classification). Multi-task learning achieves joint optimization of multiple objectives through shared feature representations, which can improve model efficiency and generalization ability.

Yang et al. trained 14 CNN models on 6, 270 white-light endoscopic images (from 516 patients) and constructed a multi-task framework that simultaneously predicted gastric cancer/benign lesions, gastric ulcer/ulcerative carcinoma, early cancer/non-cancer, *H. pylori* infection, and lymph-node metastasis (Yang et al., 2024). EfficientNetB7 achieved 83.33% accuracy (AUC 0.910) for *H. pylori* infection classification and 96.40% accuracy (AUC 0.996) for gastric cancer differentiation, and InceptionV3 achieved 79.44% accuracy for lymph-node metastasis prediction (Yang et al., 2024).

#### 2.1.4 Multicenter fusion architecture and prospective real-time validation

The transition from retrospective analysis to prospective clinical research represents a critical step in the clinical translation of AI-based endoscopic technology; early studies were predominantly single-center, retrospective validations on static images, which deviate significantly from real-world scenarios. In terms of multicenter fusion, a deep convolutional neural network with a voting-fusion strategy for gastric multi-site images effectively classified *H. pylori* infection status (uninfected, past infection, current infection), achieved diagnostic performance superior to expert endoscopists, and mitigated the bias associated with single-center data (Dai et al., 2025). The HOPE AI system, based on a multiple instance learning framework combined with a Vision Transformer and long short-term memory networks, was trained and validated on data from 6, 207 patients across seven Chinese medical institutions (>350, 000 endoscopic images and 197 videos); its internal-validation AUC reached 0.932 and external geographic-validation AUCs across seven centers ranged from 0.855 to 0.971, with sensitivity (85.7%) significantly higher than that of senior endoscopists (68.0%). Heatmap visualization revealed high concordance between the regions of interest identified by AI and the suspicious lesions annotated by experts (Hu et al., 2025).

Prospectively, a multicenter study evaluated real-time *H. pylori* assessment using a CNN-based computer-aided decision support system for *H. pylori* (CADSS-HP) under white-light endoscopy (Shen et al., 2023), and a prospective multicenter study of 701 patients from three hospitals reported real-time prediction of *H. pylori*-affected gastric regions with an AUC of 0.918, accuracy of 86.3%, sensitivity of 86.9%, and specificity of 85.9% (Yan-Dong et al., 2025). A systematic review and meta-analysis of 16 studies showed excellent diagnostic performance of endoscopy-based AI for *H. pylori* infection in both internal validation (sensitivity 0.91, specificity 0.91, AUC 0.96) and external validation (sensitivity 0.91, specificity 0.94, AUC 0.98), outperforming both junior (AUC 0.81) and senior (AUC 0.92) clinicians (Jiang et al., 2025).

### 2.2 Vision-language models and foundation models: a paradigm shift

#### 2.2.1 Introducing vision-language models into endoscopic diagnosis

Although CNNs have shown good performance in *H. pylori* classification, they only support unimodal “image → label” reasoning and cannot incorporate clinical textual information. In contrast, VLMs can understand both images and text, enabling diagnostic reasoning that fuses visual and textual data. Sun et al. (2025) developed and validated a vision-language foundation model named MC-CLIP for fully automated three-class diagnosis of *H. pylori* infection status (current infection, past infection, and no infection) under magnetically controlled capsule endoscopy (MCCE). The model was first pre-trained on over 2.42 million MCCE image–text pairs and then fine-tuned on more than 40, 000 expert-annotated images. It can autonomously select 30 representative images per case for end-to-end analysis. In multicenter internal (n = 220) and external (n = 208) validation cohorts, MC-CLIP achieved overall accuracies of 89.6% and 86.6%, respectively, with a sensitivity for current infection as high as 91.4%. The sensitivities for past infection (83.7% internal, 72.5% external) significantly outperformed those of senior endoscopists (71.4% and 68.6%) and junior endoscopists, while maintaining a specificity above 90% (Sun et al., 2025). This model effectively overcomes the limitations of traditional CNNs that depend on single images and manual image selection, and it is especially capable of recognizing subtle mucosal changes after eradication therapy, thereby reducing missed diagnoses of past infection. In the field of general-purpose endoscopic VLMs, LLaVA-Endo, a large language and vision assistant for gastrointestinal endoscopy, addresses the poor interactivity and limited adaptability of existing AI methods that rely on standalone vision models by generating multimodal instruction datasets and applying progressive transfer learning, and it outperforms state-of-the-art methods in evaluations (Yao et al., 2024). With even greater clinical practicality, researchers developed and prospectively validated the Report-Angel system—an integrated AI system based on a multimodal large language model and traditional deep learning models for automatic generation of upper gastrointestinal endoscopy reports. Trained on 20, 617 image–text pairs, the system can process endoscopic videos in real time and produce a detailed draft report immediately after the examination. In prospective internal (n = 300) and external (n = 300) validation cohorts, the clinically acceptable draft report rates reached 79.3% and 83.3%, respectively. At the case level, report completeness was 88.51% and accuracy was 78.93%; at the lesion level, report accuracies on retrospective images, prospective single-center videos, and prospective multicenter videos were 91.92%, 89.07%, and 83.94%, respectively, with an average processing time of only 1.5 seconds per lesion (Jiang et al., 2026).

#### 2.2.2 Foundation models: large-scale pre-training reshaping the paradigm of endoscopic AI development

Foundation models learn general visual representations through large-scale self-supervised pre-training and require only a small amount of labeled data for fine-tuning to adapt to downstream tasks, thereby reshaping the development paradigm of endoscopic AI (Boers et al., 2024; Jong et al., 2026). Taking the GastroNet-5M gastrointestinal endoscopy foundation model as an example, this model integrates 4, 820, 653 endoscopic images from approximately 500, 000 procedures across eight Dutch medical centers. The foundation model developed via self-supervised learning outperforms other models on 17 endoscopic AI tasks (including classification and segmentation) (Jong et al., 2026). Furthermore, a comparison of three self-supervised frameworks (SimCLRv2, MoCo v2, and DINO) showed that in-domain self-supervised pre-training with the DINO framework significantly surpassed any natural-image-based pre-training method, achieving superior downstream task performance and greater robustness (Boers et al., 2024).

#### 2.2.3 Limitations of vision-language models and foundation models

Although vision-language models and foundation models have approached or even surpassed expert-level performance in fields such as medical imaging, limitations remain before their clinical adoption. Although medical vision-language foundation models possess expert-level chest radiograph diagnostic capabilities, they exhibit systematic underdiagnosis bias against marginalized groups such as women, Black people, and Black women, with fairness gaps significantly larger than those of practicing radiologists, creating risks of inequitable deployment (Yang et al., 2025). Although they can be flexibly adapted across tasks, modalities, scanners, acquisition processing, and institutions without large-scale retraining or parameter updates, they still face challenges including sensitivity, insufficient standardization/automation, difficulty in semantic alignment, heterogeneous data, privacy/label leakage, computational power, clinical integration, and human-machine collaboration (Yang et al., 2026). Federated learning mitigates privacy and data governance issues by not exchanging raw data, but risks such as model inversion, gradient leakage, and adversarial attacks remain; mechanisms such as differential privacy, encryption, secure authentication, and traceable accountability are needed, and the trade-off between privacy protection and model performance must be balanced (Rieke et al., 2020). Informed consent, data ownership, algorithmic accountability, transparency, and automation bias all require clear regulatory frameworks and continuous post-deployment monitoring. The lack of ethical safeguards and regulation can endanger human rights, social equity, and public trust, while traditional ethical review mechanisms struggle to address new AI risks (World Health Organization, 2026). A South African study found that large language models are regarded as beneficial but risky tools, consistently emphasizing that ethical review should “augment rather than automate, “ and advocating human-centered governance grounded in contextualized and community values (Obasa and Kabanda, 2026).

### 2.3 Generalization and data bias: core challenges in clinical translation

#### 2.3.1 Cross-scenario generalization: from academic centers to primary hospitals

Whether the excellent performance of AI models on training data can be transferred to real-world clinical settings with limited resources is a core question determining their clinical value. One study developed and implemented an AI model from rural to regional medical centers for automatic assessment of *H. pylori* infection and gastric precancerous lesions based on routine endoscopic images. During the model implementation phase, the diagnostic accuracy of AI for *H. pylori*, CAG, and intestinal metaplasia reached 91.3%, 79.9%, and 63.4%, respectively, and the AI-predicted prevalence showed no significant difference from clinical observations (Chiang et al., 2026). However, most current endoscopic AI systems are trained on curated, high-quality images from advanced centers. In community medical centers, endoscopic image quality and equipment settings differ substantially, leading to a marked decrease in AI performance in real-world practice. Even minor image perturbations can cause model errors, making it difficult to identify poorly visualized lesions (De Groof, 2024).

#### 2.3.2 Endoscopic equipment settings and image bias

The image processing enhancement settings of endoscopic equipment represent an often-overlooked but profoundly influential source of bias. After retraining using image enhancement-based data augmentation, two AI systems—one for Barrett’s esophagus neoplasia detection (CADe) and one for colorectal polyp diagnosis (CADx)—showed substantially reduced variability in performance under different post-processing enhancement settings: sensitivity and specificity variability decreased to 2% and 1% for CADe, and to 2% and 8% for CADx, respectively (Jong et al., 2025). While AI plays an important role in medical image analysis, differences between equipment from different manufacturers lead to a “domain shift” problem. To address this, “domain adaptation” techniques transform images to effectively improve the generalizability and reading accuracy of AI models (Kim et al., 2023). Kim et al. (2025) proposed a dual-backbone architecture that integrates a general-purpose vision foundation model (DINOv2) with a domain-specific endoscopy model (GastroNet), achieving state-of-the-art performance (Recall@1: 97.71%, Recall@5: 99.14%, mean average precision: 96.74%), significantly outperforming single-backbone baseline models.

#### 2.3.3 Interference of *H. pylori* infection on AI-based gastric tumor detection and the elevation of clinical evidence levels

*H. pylori* infection-induced mucosal inflammatory changes not only increase the difficulty of diagnosing *H. pylori* itself but may also interfere with AI-based detection of gastric tumors. A study evaluating the impact of *H. pylori* infection status on deep learning-assisted white light endoscopic detection of gastric tumors found that mucosal inflammatory changes in *H. pylori*-positive patients led to an increased false-positive rate of AI, with overall accuracy dropping to 85.0%; eradication of the infection restored diagnostic accuracy (Yan et al., 2026).

The clinical translation of AI endoscopic technology requires a progressive upgrade from retrospective validation to prospective randomized controlled trials (RCTs). Regarding RCT evidence for endoscopic AI, the WISENSE system significantly reduced blind spots during esophagogastroduodenoscopy (EGD), establishing RCT evidence standards for the clinical implementation of endoscopic AI (Wu et al., 2019). Subsequently, the same team used a deep learning-based RCT for gastric tumor detection to further demonstrate that AI assistance can significantly reduce the missed diagnosis rate of gastric tumors (Wu et al., 2021). Recently, an RCT involving the AI Endoscopy Quality Control Assistant System (EQCA) across 32, 017 patients in seven hospitals demonstrated that real-time AI quality control significantly improves the detection rate of upper gastrointestinal tumors and precancerous lesions (Chen et al., 2025). It should be emphasized, however, that these landmark RCTs concern other upper-gastrointestinal AI applications—examination-quality control and gastric tumor detection—rather than *H. pylori* management itself, and therefore do not constitute direct randomized evidence for AI-assisted *H. pylori* care. Prospective RCTs targeting AI-based endoscopic diagnosis of *H. pylori* remain insufficient, and there is an urgent need to introduce RCT evidence standards using hard endpoints such as *H. pylori* eradication rate or gastric cancer incidence.

### 2.4 Practical considerations for clinical integration of endoscopy AI

Although the diagnostic performance of endoscopy AI continues to improve, its clinical value ultimately depends on workflow integration, clinician training, false-positive burden, human–computer interaction, and regulation—factors that determine whether benchmark accuracy translates into patient benefit. Seamless integration with existing endoscopy and reporting systems is essential: AI is most useful when it operates in real time without disrupting the examination, while automated documentation combined with AI-driven quality monitoring (e.g., withdrawal-time tracking and feedback on examination completeness) can reduce the burden. However, interoperability with electronic health records and hospital IT infrastructure remains limited, particularly in resource-constrained settings (Chen et al., 2025; Wu et al., 2019; Yagnik et al., 2026).

The use of AI also requires endoscopists to be trained not only in operating the system but also in understanding its limitations, biases, and appropriate use cases; AI assistance may even accelerate trainees’ skills by reinforcing visual recognition and classification (Yagnik et al., 2026). False-positive alerts distract endoscopists, prolong procedures, and contribute to alarm fatigue, while inflammatory and post-eradication mucosal changes can themselves increase false-positive rates and reduce overall accuracy (Yagnik et al., 2026; Yan et al., 2026). Finally, clinical deployment requires clear regulatory pathways, transparent handling of algorithm updates, post-market surveillance, and a clear definition of medicolegal accountability for AI-assisted decisions; current frameworks remain fragmented, and many approved devices do not clearly document their AI/ML functions (De Groof, 2024; Yagnik et al., 2026).

## 3 Precision risk stratification based on multimodal AI: the integration of endoscopy, electronic health records, and genomics

Although a single endoscopic image can effectively determine the infection status of *H. pylori*, it cannot answer whether the infecting strain is drug-resistant, nor can it predict whether the patient will progress to gastric cancer. Addressing these questions requires the integration of heterogeneous data sources, including clinical texts from electronic health records (EHRs), drug-resistance mutations from genomics, and host genetic variants. Multimodal AI represents the technical pathway to achieve such integration; however, its outputs must be applied under clinician oversight, with the human retaining responsibility for the final decision, and the underlying genomic components remain at an early stage of development.

### 3.1 Theoretical foundations and technical architectures of multimodal fusion

Multimodal learning aims to jointly learn complementary information from heterogeneous data sources, thereby effectively integrating heterogeneous features across different modalities. Based on the stage at which fusion occurs, the primary strategies can be categorized into four types: early fusion, late fusion, intermediate fusion, and Transformer-based fusion (Kumar et al., 2024). In precision diagnosis and treatment of *H. pylori*, endoscopic images, EHR text data, and genomic data are highly heterogeneous; therefore, late fusion and intermediate fusion are more suitable. A research team has developed a weakly supervised multimodal deep learning algorithm that integrates pathological whole-slide images and molecular profile data to predict patient outcomes (Chen et al., 2022b); for example, Pathomic Fusion models cross-modal feature-pair interactions using the Kronecker product and regulates the representational capacity of each modality via a gated attention mechanism, achieving end-to-end multimodal fusion of histology images and genomic data to predict survival outcomes in an interpretable manner (Chen et al., 2022a). By integrating diverse data sources such as medical imaging, pathology, EHRs, and multi-omics, and by employing robust fusion strategies and interpretable models, multimodal AI can improve diagnostic accuracy and prognostic prediction (Zhang et al., 2025). It is necessary here to distinguish between multimodal AI and agentic AI: multimodal AI refers to systems that perform joint representation and reasoning over heterogeneous data modalities (Baltrusaitis et al., 2019), whereas agentic AI refers to systems capable of autonomously planning and executing multi-step tasks and invoking external tools (Abdollahi et al., 2026; Collaco et al., 2026). Multimodal perception and reasoning are prerequisites for agentic AI, while agentic AI can be regarded as a natural extension of multimodal AI, in which perception and reasoning are coupled with autonomous action (Ma et al., 2026; Yu et al., 2026).

### 3.2 Genomics and AI-driven prediction of *H. pylori* drug resistance: from molecular mechanisms to clinical decision-making

**Table 3** compares conventional genotypic prediction with machine-learning (ML) approaches for *H. pylori* antibiotic resistance. Globally, primary and secondary resistance to clarithromycin, metronidazole, and levofloxacin has reached alarming levels (≥15% in most regions), and clarithromycin resistance markedly increases the risk of treatment failure (OR = 6.97). Genotypic testing of specific 23S rRNA and *gyrA* mutations can predict clarithromycin and levofloxacin resistance with nearly 100% accuracy (Martínez-Martínez et al., 2026; Savoldi et al., 2018), whereas known metronidazole (*rdxA*/*frxA* inactivation) and amoxicillin (PBP mutations) mechanisms explain only part of the phenotypic resistance, suggesting unknown mechanisms (Fang et al., 2024; Rocha et al., 2025; Vieni et al., 2025). Because single-nucleotide polymorphism (SNP) testing lacks sensitivity in complex resistance scenarios, ML combined with whole-genome sequencing (WGS) offers a promising alternative: systematic comparisons of ML methods identified resistance-associated mutations (Ren et al., 2022). A support vector machine achieved 100% sensitivity and specificity for clarithromycin resistance (Yu et al., 2023), and an interpretable XGBoost–SHAP model based on SNP and gene presence/absence features predicted resistance (Wang et al., 2025). Beyond genomics, multimodal EHR data (time series and clinical text) can predict antimicrobial resistance and are potentially applicable to *H. pylori* (Hardan et al., 2024; Nigo et al., 2024), and the *H. pylori* AI-Clinician system (isDQN, trained and validated on 38, 049 patients) achieved a 94.1% success rate for AI-recommended regimens versus 88.1% for non-AI regimens, replicated in an external cohort (n = 7, 186) (Higgins et al., 2025). Nevertheless, WGS-based prediction is currently constrained by turnaround time, cost, and limited availability, and genomic AI in *H. pylori* remains at an early, proof-of-concept stage; it is therefore presented here as an emerging direction requiring prospective validation rather than an established clinical tool, and its linkage to the other modules is prospective.

**Table 3:** Key AI approaches for predicting antibiotic resistance in *H. pylori.*

Antibiotic	Main resistance genes/mutations	Conventional genotypic prediction accuracy	AI/ML method	Data input	Performance improvement	Reference
Clarithromycin	23S rRNA (A2142G, A2143G)	Nearly 100%	SVM	Whole-genome sequencing (SNPs)	100% sensitivity and specificity	(Yu et al., 2023)
Levofloxacin	gyrA (N87K, D91N, etc.)	Nearly 100%	Explainable XGBoost + SHAP	WGS SNPs and gene presence/absence	High accuracy with interpretable contribution of resistance features	(Wang et al., 2025)
Metronidazole	rdxA, frxA (loss of function)	Only partially explained	Ensemble ML model comparison	Whole-genome sequencing	Systematic identification of novel resistance features beyond single-gene testing	(Ren et al., 2022)
Comprehensive regimen	—	—	Deep Q-learning (isDQN)	38, 049 patient records	AI-recommended regimen success rate 94.1% vs. non-AI 88.1%; externally validated	(Higgins et al., 2025)

### 3.3 Precision prevention and stratified management of gastric cancer driven by multi-omics integration

The carcinogenic process of *H. pylori* infection is not a linear pathway but a multi-dimensional interactive network involving the microbiome, host genome, epigenetics, and the immune microenvironment (Guo et al., 2020; Park et al., 2022). A transcriptomic risk score based on 11 gastric cancer-related genes shows a dose–response relationship with gastric cancer risk and interacts with *H. pylori* infection, with high-scoring individuals deriving greater benefit from eradication therapy (Yin et al., 2025), and *H. pylori* can activate the Hippo–YAP1 signaling pathway through GNB4 promoter demethylation, providing a candidate biomarker for early warning of gastric cancer (Liu et al., 2023). The integration of AI with multi-omics data is beginning to shift gastric biopsy from traditional descriptive histology toward data-driven precision gastroenterology (Alwahaibi, 2026); however, this integration remains largely exploratory and is presented here as a future direction rather than a mature clinical capability.

## 4 AI-assisted *H. pylori* vaccine design: from reverse vaccinology to deep learning

The continuously rising rate of antibiotic resistance is profoundly affecting the eradication strategies for *H. pylori*. As the ultimate tool for preventing infection at its source, vaccine development is becoming increasingly urgent. However, after more than 30 years of exploration, no commercial *H. pylori* vaccine is available. AI and ML are reshaping the entire vaccine design pipeline—from antigen screening and structure prediction to immunogen optimization—offering the potential to overcome the difficulties faced by traditional development paradigms.

### 4.1 Urgency and traditional challenges in *H. pylori* vaccine development

*H. pylori* vaccine research has spanned more than 30 years, with multiple clinical trials failing to meet their expected endpoints. (Zeng et al., 2015) published a phase III trial of an oral recombinant *H. pylori* vaccine in Chinese children in *The Lancet*, showing that among *H. pylori*-negative children the vaccine demonstrated 71.8% protective efficacy with a good safety profile. However, in a phase I/II trial of a three-antigen vaccine (VacA, CagA and NAP) conducted by Malfertheiner et al (Malfertheiner et al., 2018), although the vaccine robustly induced high levels of humoral immune responses against CagA, VacA and NAP, it did not confer additional protection against *H. pylori* infection following challenge with CagA-positive strains. *H. pylori* infection possesses high genetic diversity and potent immune evasion capacity (e.g., virulence factors such as γ-glutamyl transpeptidase and VacA can suppress T-cell immunity), making it difficult for traditional vaccines to elicit durable protection (Hasanzadeh Haghighi et al., 2024; Sutton and Boag, 2019). Most existing vaccine candidates remain in preclinical or early phase I stages, and the only oral urease vaccine that had entered phase III has also been discontinued. Future mainstream directions include: multi-epitope/multivalent vaccines, development of novel mucosal adjuvants and delivery systems, exploration of mRNA vaccine technology, and “anti-disease” rather than “anti-bacterial” immunomodulation strategies (Hasanzadeh Haghighi et al., 2024). To date, no *H. pylori* vaccine has been commercialized; only a UreB vaccine once entered phase III clinical trials (Liu et al., 2025b). AI-driven vaccine design replaces the traditional “trial-and-error” experimental model with computational prediction, potentially shortening the discovery cycle and reducing the risk of clinical failure, thereby addressing challenges in *H. pylori* vaccine development such as mucosal delivery, antigen conservation, and immune evasion.

### 4.2 Computational immunology and reverse vaccinology: paradigm evolution from genome to epitope prediction and the empowerment by machine learning

Reverse vaccinology overturns the traditional vaccine development paradigm that relies on pathogen cultivation by starting from pathogen genome sequences and computationally predicting candidate antigens without the need for *in vitro* culture (Rappuoli, 2000). In *H. pylori* vaccine design, immunoinformatics tools have enabled systematic screening from whole genomes to vaccine candidates: candidate epitopes have been selected from five highly conserved virulence factors with strong human leukocyte antigen (HLA) binding affinity, high population coverage (>90% in Southeast Asia), and no predicted allergenicity/toxicity (Chacon et al., 2025), and a multi-epitope self-amplifying RNA vaccine has been designed *in silico* with favorable predicted stability and immunogenic potential (Razzak et al., 2025). A minority of candidates have progressed to experimental validation—for example, a lipid nanoparticle-delivered multi-epitope vaccine (Jebali et al., 2024) and chitosan-nanoparticle-encapsulated pDNA/recombinant-protein vaccines that induced gastric mucosal SIgA and specific IgG in BALB/c mice (Amaral et al., 2025). ML further improves prediction accuracy, from the initial use of support vector machines to distinguish bacterial protective antigens from non-antigens with 92% accuracy (Bowman et al., 2011) to current applications in B-/T-cell epitope recognition and correlates-of-protection analysis (Bravi, 2024). Most *H. pylori* immunoinformatics studies nevertheless remain purely computational; the full cycle of computational prediction → *in vitro* validation → animal models → clinical trials is still a work in progress.

### 4.3 Deep learning reshapes next-generation vaccine design: from panoramic optimization to intelligent creation

Deep learning is driving a paradigm shift in next-generation vaccine design. At the application level, bioinformatics and AI are accelerating mRNA vaccine development—from sequence design and neoantigen prediction to lipid nanoparticle (LNP) formulation optimization (Imani et al., 2024). At the structural level, protein language models and structure-prediction tools such as AlphaFold2/3 rapidly resolve antigen three-dimensional conformations, epitope characteristics, and antigen–antibody interactions (Abramson et al., 2024; Jumper et al., 2021). A breakthrough in generative AI (e.g., RFdiffusion) has enabled a qualitative leap from “predicting known antigens” to “creating *de novo* immunogens” by designing diverse functional proteins with experimentally validated accuracy (Watson et al., 2023), and deep learning achieves 96.9% classification accuracy in adjuvant prediction (Ji et al., 2025). For *H. pylori*, RFdiffusion could in principle design *de novo* protein immunogens targeting conserved *H. pylori* epitopes to circumvent the immune evasion of natural antigens; however, no such design has yet been experimentally validated in *H. pylori*, and this remains a conceptual proposition.

### 4.4 Future AI directions for *H. pylori* vaccine design

AI-driven synergistic multi-antigen design must simultaneously optimize protection and safety: ML can be used to screen conserved and highly immunogenic antigen combinations (e.g., core conserved regions of UreB, CagA, and VacA), and multi-objective optimization can balance immune coverage and autoimmune risk. AI can assist in designing delivery strategies that target mucosal immunity: optimizing nanoparticle surface modifications: chitosan (Amaral et al., 2025), LNP (Imani et al., 2024), simulating oral/intranasal delivery processes, and screening adjuvants through AI (Ji et al., 2025). Novel nanovaccines (RA-NVs) have successfully induced potent mucosal (IgA) and systemic (IgG) immune responses via intramuscular injection, significantly reducing *H. pylori* colonization and inflammation, and providing a safe and effective strategy against mucosal pathogens (Xu et al., 2025).

The major shortcoming of current *H. pylori* immunoinformatics research is the lack of *in vivo* validation; most studies rely solely on computational prediction and molecular docking, with only a few conducting animal experiments. Establishing a closed loop of “computational prediction → *in vitro* validation → animal models → clinical trials” is of critical importance. The study by (Zhong et al., 2025) on AI-designed antibody-engineered probiotics targeting urease offers an interesting paradigm innovation: AI-designed antibodies delivered to the stomach by engineered probiotic EcN (engineered *Escherichia coli* Nissle 1917) significantly reduced *H. pylori* colonization, promoted mucosal repair, and did not disturb the intestinal microbiota, opening a new direction at the intersection of AI and synthetic biology in *H. pylori* prevention and treatment.

## 5 Digital patient management: new media and AI for enhancing eradication rates and adherence

**Table 4** summarizes the impact of various digital interventions on the *H. pylori* eradication rate and adherence, illustrating both conventional digital health tools and AI-driven strategies. It is important to distinguish conventional digital health tools—such as short-message, telephone, and WeChat/social-media reminders, which are not inherently AI-based—from genuinely AI-driven interventions, such as reinforcement-learning-optimized personalized messaging and generative voice cloning, which are discussed separately below. In *H. pylori* eradication therapy, even when precise diagnosis and resistance prediction are available, poor adherence remains an independent risk factor for eradication failure and may induce antibiotic resistance (Chey and Wong, 2007). The European Hp-EuReg registry, analyzing over 38, 000 patients, shows that overall adherence to eradication therapy in clinical practice is relatively good (non-adherence rate only 1.7%). However, factors such as uninvestigated/functional dyspepsia, rescue treatment, longer treatment duration (10/14 days), occurrence of adverse events, and non-bismuth sequential/concomitant regimens are significantly associated with non-adherence, while full adherence is the strongest predictor of treatment success (Huguet et al., 2024). To improve adherence, a variety of digital interventions have demonstrated evidence-based value. Twice-daily short-message re-education can improve the eradication rate in younger patients and significantly enhance treatment adherence (Wang et al., 2019). Telephone-based re-education also significantly increases the eradication rate without increasing adverse reactions (Zhao et al., 2020); however, in a study conducted in China, daily telephone re-education did not significantly improve the eradication rate or adherence, but effectively reduced adverse events (Wang et al., 2015). Semi-automated intensive follow-up based on clinical databases can significantly improve the eradication rate (especially in intention-to-treat analysis) and treatment adherence (Chen et al., 2021).

**Table 4:** Impact of digital interventions on *H. pylori* eradication rate and adherence.

Intervention	Study design	Main findings (eradication rate/adherence)	Features	Reference
Twice-daily short-message re-education	RCT	Improved eradication rate and adherence in younger patients	Low cost, automated reminders	(Wang et al., 2019)
Telephone re-education	RCT	Increased eradication rate without additional adverse events	Real-time communication	(Zhao et al., 2020)
WeChat platform health education	Meta-analysis (13 studies, 2, 942 patients)	Significantly improved eradication rate and adherence; no increase in adverse events	Highly interactive, personalized	(Fan et al., 2025)
WeChat mini-program self-monitoring	Multicenter RCT	Proactive monitoring, improved adherence and eradication rate	Upgraded from passive reminders to active management	(Sun et al., 2022)
Reinforcement learning-based personalized SMS	AI intervention study	Significantly improved medication adherence	AI dynamically optimizes intervention timing and content	(Lauffenburger et al., 2024)
AI voice-cloned health education	AI comparative study	Improved knowledge acquisition, adherence, and psychological well-being compared with traditional/physician voice	Patient’s own voice enhances trust	(Sun et al., 2026)

Using social media platforms such as WeChat can significantly enhance patients’ disease knowledge, medication adherence, and *H. pylori* eradication rate (Luo et al., 2020; Ma et al., 2021). A meta-analysis including 13 studies with 2, 942 patients further confirmed that new media education significantly improves eradication rates and patient adherence without increasing adverse events, with personalized and interactive communication platforms, especially WeChat-based interventions, exerting particularly prominent influence (Fan et al., 2025). Building on this, multicenter randomized controlled trials of WeChat mini-programs (Sun et al., 2022) and daily management models (Yang et al., 2023) have upgraded passive reminders to active patient self-monitoring, significantly improving both adherence and eradication rates. Studies on WeChat-based doctor-patient interaction have further verified its role in enhancing eradication rates, medication adherence, and treatment satisfaction (Huang et al., 2026).

The introduction of AI adds a new dimension to adherence management: personalized short message interventions based on reinforcement learning can significantly improve medication adherence (Lauffenburger et al., 2024), and AI-assisted health education using the patient’s own voice cloning outperforms traditional education or doctor voice cloning in improving knowledge acquisition, treatment adherence, and psychological well-being (Sun et al., 2026). In conclusion, digital patient management, especially interventions based on new media (e.g., WeChat) and AI (e.g., personalized short messages, voice cloning), can significantly improve patient adherence and eradication rates in *H. pylori* eradication therapy.

## 6 Challenges and prospects

### 6.1 Data bias and fairness: multi-level challenges spanning data, models, and personnel

Cross-scenario performance decay in AI models constitutes the primary barrier to clinical deployment, rooted in bias across three levels: data, models, and personnel (Ricci Lara et al., 2022). In endoscopic AI for *H. pylori*, transferring a model from remote areas to medical centers led to an overall decline in the diagnostic accuracy for *H. pylori* infection, CAG, and intestinal metaplasia, revealing severe geographic bias and underscoring markedly insufficient robustness to low-quality primary-care images and atypical mucosal changes (Chiang et al., 2026). The status quo of fairness evaluation is equally concerning: current research on fairness in medical AI generally suffers from key issues including a disconnect between technology and clinical practice, a scarcity of high-quality studies, a limited scope of bias attributes considered, an over-reliance on group fairness metrics, and insufficient engagement from clinicians (Liu et al., 2025a). Furthermore, medical AI models often exhibit substantial performance drops in cross-distribution testing (e.g., AUC plummeting from 0.95 to 0.63) due to systematic biases inherent in public datasets, such as sampling bias, annotation subjectivity, label noise, and natural language processing-generated pseudo-labels. A paradigm shift through the construction of diverse datasets and the implementation of bias mitigation strategies is urgently required (Ramadan et al., 2026).

### 6.2 Challenges and pathways for the clinical translation of precision diagnosis and treatment AI for *H. pylori*

Numerous AI models deliver excellent performance on retrospective data yet lack validation through prospective randomized controlled trials (RCTs). Although the field of endoscopic AI has benchmark RCTs such as WISENSE (Wu et al., 2019) and EQCA (Chen et al., 2025)—which, as noted above, evaluate other upper-gastrointestinal applications rather than *H. pylori* management—prospective RCTs remain absent for *H. pylori* endoscopic diagnosis, drug resistance prediction, and AI-driven clinical decision support (AI-Clinician). Only an RCT comparing AI-assisted versus standard diagnosis and treatment with the eradication rate as the primary endpoint can bridge this evidence gap. Uncertainty also persists at the regulatory level: among 64 FDA-approved AI/ML medical devices, only 45% had official announcements explicitly mentioning AI/ML-related terminology, with the majority concentrated in radiology and cardiology (Benjamens et al., 2020). To ensure the long-term safety and effectiveness of clinical ML/AI algorithms, an “AI Quality Improvement (AI-QI)” unit should be established within hospitals to take charge of continuous algorithm monitoring and updating (Feng et al., 2022). Addressing these challenges necessitates deep multidisciplinary integration encompassing gastroenterology, microbiology, genomics, and AI engineering, establishing innovative paradigms such as a trimodal fusion of “endoscopy + WGS + EHRs”. Forming cross-disciplinary teams composed of clinicians, bioinformaticians, AI engineers, and microbiologists provides the organizational guarantee for successful clinical implementation.

A practical implementation timeline can be staged as follows. In the near term (1–3 years), hospitals can deploy or evaluate endoscopic assistance, structured EHR-based adherence support, and registry-based monitoring within existing governance; these tools still require local calibration and prospective safety evaluation. In the medium term (3–5 years), targeted resistance testing and multimodal decision support may be evaluated in high-risk, recurrent, or treatment-failure populations when laboratory turnaround and reimbursement are acceptable. In the longer term (5–10 years), WGS–ML, host multi-omics, and AI-designed vaccine candidates may enter broader clinical studies only if they demonstrate reproducibility, cost-effectiveness, equity, and improved patient outcomes. These intervals are proposed milestones rather than forecasts, because regulatory review, data infrastructure, and prospective evidence may accelerate or delay implementation.

### 6.3 Future directions: multimodal synergy and a full-chain evidence-based AI system

Breakthroughs in endoscopic AI and precision diagnosis and treatment of *H. pylori* will proceed along five mutually reinforcing directions. First, a unified platform driven by foundation models will upgrade visual foundation models such as GastroNet-5M (Jong et al., 2026) into multimodal foundation models. Using large-scale endoscopic vision pre-training as its backbone, it could connect to downstream tasks including EHR text, targeted resistance prediction, and gastric cancer risk stratification, while allowing missing modalities. Med-PaLM M provides an architectural blueprint, but not evidence of *H. pylori* clinical readiness (Tu et al., 2024). Second, federated learning could enable multiple parties to collaborate by exchanging model parameters rather than raw data, protecting privacy and reducing data silos; its usefulness for *H. pylori* still requires disease-specific validation (Puca et al., 2025; Rieke et al., 2020). Third, genome-transcriptome-proteome representation learning could improve biological discovery and resistance surveillance, but it should remain a research capability until genotype–phenotype concordance, reproducibility, turnaround, and cost are established in clinically relevant cohorts (Lin et al., 2023b). Fourth, prospective multicenter clinical trials should test discrete use cases: AI-assisted endoscopic diagnosis versus standard endoscopy, AI-supported regimen selection versus usual care, and AI-supported follow-up versus standard follow-up (Cruz Rivera et al., 2020). Primary endpoints should include diagnostic accuracy, eradication rate, adherence, adverse events, and downstream resource use, with prespecified subgroup analyses by age, region, and resistance prevalence (Lekadir et al., 2025). Fifth, real-world evidence and AI quality improvement should provide continuous post-deployment monitoring through *H. pylori* registries, drift detection, subgroup fairness checks, and controlled model updating (Feng et al., 2022; Huguet et al., 2024). Together, these directions define a staged path from technical feasibility to clinical utility rather than implying that all components are ready for simultaneous implementation.

## 7 Conclusion

The management of *H. pylori* infection demands a shift from empirical strategies to data-driven precision care. We have proposed a conceptual five-module framework—resistance epidemiology, multimodal AI diagnosis, AI-driven resistance prediction and personalized therapy, AI-empowered vaccine design, and digital patient management—that integrates these elements into a unified multidisciplinary system. Key advances include the evolution of endoscopic AI from static CNNs to vision-language foundation models, the progression of genomic ML from single-SNP testing toward whole-genome resistance interpretation, and the emergence of generative AI for *de novo* immunogen design; digital adherence tools are beginning to leverage reinforcement learning to become active, personalized therapeutic components. However, this framework is proposed rather than established: most components rest on retrospective or proof-of-concept evidence, and clinical translation remains constrained by cross-scenario performance degradation, a scarcity of prospective randomized controlled trials using hard endpoints, and the absence of robust regulatory and continuous quality-improvement frameworks. The path forward requires federated privacy-preserving networks built on multimodal foundation models, multicenter RCTs validating the full “perception–reasoning–intervention” chain, and the embedding of AI within real-world evidence ecosystems to establish a precise, equitable, and continuously learning global health enterprise.
